# The Impact of Maternal Obesity on Adipose Progenitor Cells

**DOI:** 10.3390/biomedicines11123252

**Published:** 2023-12-08

**Authors:** Simon Lecoutre, Salwan Maqdasy, Mélanie Lambert, Christophe Breton

**Affiliations:** 1 Nutrition and Obesities: Systemic Approach Research Group, Nutriomics, Sorbonne Université, INSERM, F-75013 Paris, France; 2 Department of Medicine (H7), Karolinska Institutet Hospital, C2-94, 14186 Stockholm, Sweden; salwan.maqdasy@ki.se; 3 U978 Institut National de la Santé et de la Recherche Médicale, F-93022 Bobigny, France; melanie.lambert@univ-paris13.fr; 4 Université Sorbonne Paris Nord, Alliance Sorbonne Paris Cité, Labex Inflamex, F-93000 Bobigny, France; 5 Maternal Malnutrition and Programming of Metabolic Diseases, Université de Lille, EA4489, F-59000 Lille, France; 6 U1283-UMR8199-EGID, Université de Lille, INSERM, CNRS, CHU Lille, Institut Pasteur de Lille, F-59000 Lille, France

**Keywords:** perinatal period, maternal obesity, developmental origin of health and disease, epigenome, gene expression, fat expansion, adipose tissue

## Abstract

The concept of Developmental Origin of Health and Disease (DOHaD) postulates that adult-onset metabolic disorders may originate from suboptimal conditions during critical embryonic and fetal programming windows. In particular, nutritional disturbance during key developmental stages may program the set point of adiposity and its associated metabolic diseases later in life. Numerous studies in mammals have reported that maternal obesity and the resulting accelerated growth in neonates may affect adipocyte development, resulting in persistent alterations in adipose tissue plasticity (i.e., adipocyte proliferation and storage) and adipocyte function (i.e., insulin resistance, impaired adipokine secretion, reduced thermogenesis, and higher inflammation) in a sex- and depot-specific manner. Over recent years, adipose progenitor cells (APCs) have been shown to play a crucial role in adipose tissue plasticity, essential for its development, maintenance, and expansion. In this review, we aim to provide insights into the developmental timeline of lineage commitment and differentiation of APCs and their role in predisposing individuals to obesity and metabolic diseases. We present data supporting the possible implication of dysregulated APCs and aberrant perinatal adipogenesis through epigenetic mechanisms as a primary mechanism responsible for long-lasting adipose tissue dysfunction in offspring born to obese mothers.

## 1. Introduction

Childhood obesity represents a pressing global public health challenge that requires effective preventive measures [1,2]. Numerous observational studies conducted in humans have demonstrated a connection between exposure to maternal obesity during perinatal development and an increased risk of obesity and metabolic disorders later in life [3,4,5,6,7,8]. This finding aligns with the principles of the Developmental Origins of Health and Disease (DOHaD) hypothesis, which suggests that the predisposition to obesity can be influenced during fetal development [9]. The DOHaD hypothesis emphasized that improving maternal well-being can, in turn, positively impact the health of offspring. This underscores the importance of focusing on maternal metabolic health as a new, valuable clinical treatment. However, there are ethical challenges in empirically investigating the DOHaD hypothesis at the cellular and molecular levels due to restrictions related to accessing fetal tissue.

The developmental origin of obesity perpetuates a concerning cycle of metabolic diseases that persist across generations. In recent decades, there has been a two-fold increase in obesity and its related metabolic disorders, reaching epidemic proportions [10]. Primary factors contributing to the development of type 2 diabetes (T2D) include obesity and the dysfunction of white adipose tissue (WAT), which lead to insulin resistance [11]. It is important to note that insulin resistance can initiate locally within hypertrophic adipocytes long before glucose intolerance becomes apparent [12]. When WAT’s capacity for expansion is exhausted, surplus fat begins to accumulate in other tissues, such as skeletal muscle and the liver, resulting in systemic insulin resistance [13,14]. Additionally, chronic inflammation within WAT plays a pivotal role in triggering insulin resistance and T2D in individuals with obesity [11]. Indeed, the restructuring of WAT due to obesity triggers local inflammatory responses that can extend systemically, culminating in widespread insulin resistance and T2D [11,15,16].

The adaptability of WAT to an obesogenic environment, specifically its ability to sequester and buffer toxic lipids, relies on a resident pool of adipocyte progenitor cells (APCs) that can be enlisted for adipogenesis. Recent research has pinpointed the late fetal and lactation periods as critical developmental windows that establish the number and characteristics of APCs, influencing the trajectory of fat accumulation throughout childhood, adolescence, and adulthood [17,18,19]. These groundbreaking studies underscore the perinatal environment as a pivotal regulator of postnatal adiposity and adipose tissue plasticity. Nevertheless, the impact of maternal obesity on APCs within the offspring’s WAT remains largely unexplored.

In this review, we aim to provide insights into the developmental timeline of lineage commitment and differentiation of APCs and their role in predisposing individuals to obesity and metabolic diseases. We will also summarize the potential role of aberrant perinatal adipogenesis as a primary mechanism responsible for the programming of obesity and its associated complications in offspring born to mothers with obesity or diabetes during pregnancy.

## 2. Maternal Health and Determination of Metabolic Programming in Offspring

The concept of the DOHaD suggests a connection between early developmental environmental factors and the predisposition to metabolic disorders in later life. This concept posits that an inadequate nutritional environment during the perinatal period, whether due to undernutrition or overnutrition, can program or imprint the development of key tissues responsible for regulating energy balance. This programming can lead to lasting physiological changes, resulting in disrupted energy balance and the development of metabolic diseases, such as obesity and T2D [20].

The concept, originally known as the Barker hypothesis or fetal programming, originated from epidemiological studies. David Barker was the first to observe a link between intrauterine growth retardation and low birth weight, and an increased risk of metabolic syndrome-related diseases in adulthood [20]. The Dutch famine of 1944–1945 is an illustration of the offspring of famine-exposed mothers exhibiting low birth weight and a higher incidence of dyslipidemia, obesity, and T2D in later life [21]. More recently, adults born during the Chinese famine of 1959–1961 were also found to have an increased susceptibility to overweight and T2D, significantly contributing to China’s current T2D epidemic [22]. David Barker introduced the notion of a “thrifty phenotype”, highlighting the importance of development on metabolic health in adulthood. He argued that nutritionally inadequate conditions during pregnancy not only affected fetal growth but also triggered enduring alterations in insulin secretion and glucose metabolism [23]. Among individuals born with low birth weight who had rapid catch-up growth due to postnatal hypercaloric nutrition, a significant acceleration in the development of adult-onset diseases was observed [24]. As suggested by the predictive adaptive response concept, the degree of incongruity between the prenatal and postnatal environments stands as the central paradigm in developmental metabolic programming [25].

In recent decades, there has been a concerning global surge in the prevalence of obesity and T2D, overshadowing the issue of malnutrition. A growing number of children are now exposed to intrauterine conditions characterized by maternal obesity and other metabolic complications, including T2D, hypertension, and dyslipidemia [26]. Even in cases where mothers are not obese, metabolic challenges during pregnancy, such as excessive gestational weight gain and elevated maternal blood glucose levels, exert a significant impact on fetal growth and may result in long-term consequences [27]. Children born under these circumstances are at risk of facing an obesogenic environment during both childhood and adulthood. Therefore, it is crucial to conduct solid research to gain further insights into the long-lasting impacts of maternal obesity and gestational metabolic dysfunctions.

Elevated maternal blood glucose levels are associated with increased fetal growth, a condition known as macrosomia [28]. According to the Pedersen hypothesis, macrosomia results from the transplacental transport of glucose in the presence of maternal hyperglycemia, which leads to increased insulin secretion from the fetal pancreas [29]. Substantial evidence, including findings from the Hyperglycemia and Adverse Pregnancy Outcome (HAPO) study, emphasizes the pivotal role of maternal hyperglycemia. The HAPO study established a clear linear relationship between maternal glucose levels and birth weight [30]. However, it is now recognized that other factors, such as maternal obesity, also significantly contribute to the risk of obstetric complications and the promotion of excessive fetal adiposity [9,27]. Epidemiological evidence underscores a positive correlation between maternal body mass index (BMI) and the future health of offspring, particularly increasing the likelihood of cardiovascular diseases and T2D [31,32]. Children born to mothers who lost a significant amount of weight following bariatric surgery exhibit a lower prevalence of overweight and obesity and improved cardiometabolic indicators during adolescence compared to their siblings born before their mothers underwent the surgical procedure [33]. Additionally, infants with macrosomia born to mothers with gestational diabetes mellitus (GDM) face an increased risk of developing metabolic syndrome during childhood [3]. Some studies have also demonstrated that the offspring of mothers with GDM have a heightened susceptibility to obesity, T2D, and/or impaired insulin sensitivity and secretion [34]. Furthermore, a study investigated the relationship between maternal BMI and offspring adiposity, using a sample of 105 mother-infant pairs examined approximately two weeks after delivery. The study revealed significant positive correlations between maternal BMI and newborn adiposity. For each incremental increase in maternal BMI, there was an approximately 8 mL increase in WAT volume, illustrating a robust and direct connection between maternal body composition and the developmental expansion of WAT [35]. Thus, siblings born after their mother had lost weight were less obese and exhibited improved cardiometabolic risk profiles persisted into adulthood, compared to their siblings born when their mother was obese [36]. Maternal obesity may have immediate effects, such as impaired organ growth during development, while other effects, such as metabolic programming, may manifest later in response to further stimuli [37]. In mice, offspring born to females with GDM exhibit multiple metabolic dysfunctions in adulthood, including increased body weight, hyperphagia, and insulin resistance [38]. In conclusion, there is evidence supporting the notion that both maternal adiposity and glucose levels contribute to excess fat accumulation in the fetus.

Nonetheless, a significant revelation highlights the enduring influence of early postnatal overnutrition, resulting in persistent changes in energy balance and increased susceptibility to obesity and insulin resistance throughout adulthood. It is noteworthy that maternal obesity during lactation in rodents has more adverse metabolic consequences on offspring than maternal obesity during pregnancy [39]. These findings underscore the significance of early childhood as a critical developmental phase in which nutritional challenges or metabolic imbalances carry long-term consequences on body weight regulation. There is substantial evidence indicating that maternal obesity significantly affects milk composition. Obese rats produce milk with higher energy and fat content compared to lean rats. Additionally, feeding rats a Western-style diet during lactation reduces milk protein and increases milk fat [40,41,42,43,44]. In humans, maternal body adiposity is correlated with the concentration of leptin in breast milk [45,46,47,48,49]. Furthermore, both maternal serum and breast milk levels of adiponectin are inversely related to infant weight and abdominal circumference [50]. Nevertheless, the exact role played by adipokines in maternal milk in the metabolic programming of offspring remains an area of ongoing research [45]. Given the influence of obesity on adipokine levels in both plasma and breast milk, along with its ability to alter the nutrient composition of milk, it has been demonstrated that maternal obesity negatively affects breastfeeding success in humans and lactation performance in rodents [45,51]. Furthermore, the duration of lactation plays a pivotal role in metabolic programming [45]. Extending the lactation period has been found to effectively protect rat pups against diet-induced obesity in adulthood by enhancing brown adipose tissue (BAT) [52]. In contrast, early weaning has been shown to lead to increased adiposity, insulin resistance, and dyslipidemia in adult rats [53].

The adverse consequences of maternal obesity seem to become apparent during developmental periods where epigenetic processes are highly dynamic and sensitive to environmental cues. The most sensitive period for epigenetic programming of WAT is during adipogenesis, which marks the differentiation of APCs into mature adipocytes [9]. However, it is important to note that the timing of this critical window can vary among different species. Although there is no definitive evidence establishing a direct link between maternal obesity and predisposition to adiposity in offspring, different animal studies have provided valuable insights into the potential mechanisms underlying this connection [37,54].

## 3. Adipose Tissue Organogenesis

### 3.1. Role of Adipose Tissue

Adipocyte roles extend beyond their ability to store excess calories as fat in large droplets. Although this energy storage is advantageous from an evolutionary perspective, it is a feature exclusive to vertebrates [55]. Besides their role as energy depots, adipocytes offer various other vital functions. For instance, in colder climates, animals tend to have more adipose tissue, which provides better insulation and the ability to generate heat [56]. Adipocytes also act as cushions, particularly in areas exposed to mechanical stress, like the palms, buttocks, and heels [11]. Internally, adipose tissue cushions vital organs such as the heart, adrenal glands, kidneys, and ovaries. Furthermore, in warm-blooded mammals, there is a distinct class of adipocytes, called brown or beige/brite adipocytes, which originates from the browning of subcutaneous WAT, which plays a key role in energy expenditure and heat generation [57]. Adipocytes respond to the body’s signals by either storing or releasing nutrients upon requirement. During times of nutrient shortage, where blood glucose levels drop, hormones like glucagon or noradrenaline stimulate adipocytes. These signals activate lipases, enzymes responsible for breaking down stored fats, resulting in the release of non-esterified fatty acids and glycerol from lipid droplets into the bloodstream [58]. These released nutrients are then utilized by organs like the liver and skeletal muscles. Conversely, during nutrient abundance, insulin inhibits lipases and promotes glucose uptake and de novo lipogenesis [58]. These lipids are then stored in lipid droplets to save the excess nutrients for later use. During obesity-related metabolic decline, genetic and epigenetic modifications leading to increased collagen and inflammatory cytokine production, cell senescence, and death restrain the capacity of adipocytes to effectively respond to various external signals, particularly insulin [11,15,16,59,60]. This leads to higher levels of glucose and lipids in the bloodstream.

### 3.2. Adipose Tissue Development

The primary development of adipose tissue and adipogenesis occurs during the critical phases of pregnancy and lactation, during which alterations in the perinatal environment likely imprint lasting changes in the characteristics of offspring adipose tissue. In rodents, adipogenesis is active during the perinatal period, primarily during the last week of gestation. The initial appearance of fat cells is observed between the 14th and 18th days of gestation, and this process intensifies during early postnatal life, extending until puberty [17,18,61,62,63]. Subcutaneous inguinal adipocytes emerge during the perinatal period, while most intra-abdominal depots develop after birth [64]. It is noteworthy that more than half of visceral adipocytes and over 20% of subcutaneous adipocytes in adult mice emerge from the differentiation of APCs during the early pubertal period, specifically between postnatal days 18 and 34 [18].

In humans, adipogenesis and development of adipose tissue predominantly occur before birth. The onset of adipogenesis typically begins around the 14th to 17th week of gestation, initially forming clusters of fat lobules [65]. This process initiates in the head and neck region, subsequently extending to the trunk and later to the limbs [65,66]. By the 28th week of gestation, fat depots become more organized [65]. Following birth, both the number and size of adipocytes increase. In a cross-sectional study involving children, adipocyte size reaches adult levels between 6 months and 1 year of age and then gradually decreases between one and two years [67]. The number of adipocytes experiences another surge during adolescence [68]. Therefore, the quantity of adipocytes within a specific depot is predominantly established during early life and remains relatively constant throughout adulthood [68]. However, recent studies suggest that adipogenesis may indeed contribute to the expansion of adipose tissue through hyperplasia in adults, albeit with notable regional variations [69]. BAT was initially documented in 1551 by Conrad Gesner, a Swiss naturalist, in the interscapular region of marmots. In 1902, when Hatai first described BAT in human embryos, it was referred to as “an interscapular gland corresponding to the so-called hibernating gland of lower animals” [70]. In contrast to WAT, BAT tissue emerges before WAT and is readily identifiable at birth in most mammals [71].

### 3.3. Adipogenesis

Adipocytes originate from multipotent mesenchymal stem cells (MSCs). Adipogenesis commences with MSCs committing to the adipogenic lineage, followed by their differentiation into preadipocytes [72,73,74]. These preadipocytes then undergo terminal differentiation to become mature adipocytes, capable of storing lipids in large monolocular lipid droplets and exhibiting endocrine functions. In contrast to our relatively limited understanding of the commitment phase, it is apparent that the adipogenic commitment process involves the integration of various pro- and anti-adipogenic growth-factor signals. Canonical WNT pathways (especially WNT5, WNT6, WNT10a, and WNT10b), TGFβ, and platelet-derived growth-factor (PDGF) signaling have been demonstrated to inhibit adipogenesis [72,73,75]. On the other hand, pro-adipogenic signals encompass insulin, bone-morphogenic-protein (BMP) signaling (especially BMP2 and BMP4), and extracellular matrix (ECM) composition and associated stiffness [11,73]. For instance, BMP2 and BMP4 activate SMAD4 and its heterodimeric partners. Activated SMAD4, in turn, promotes terminal differentiation in preadipocytes by stimulating the transcription of PPARγ [76]. Notably, BMP4 induces the nuclear entry of the PPARγ transcriptional activator, zinc-finger protein 423 (ZFP423) [77], by dissociating an intracellular protein complex between Wnt1-inducible-signaling pathway protein 2 (WISP2) and ZFP423. This complex dissociation allows ZFP423 to enter the nucleus, thus activating PPARγ transcription and committing precursor cells to the adipocyte lineage [77]. The activation of PPARγ stimulates the expression of numerous genes defining terminally differentiated adipocyte phenotypes [78,79,80]. During adipogenesis, epigenetic mechanisms are thought to be crucial in orchestrating the activity of transcription factors and chromatin conformation changes in the developmental program of APCs [74,78,81,82,83,84]. Epigenetic mechanisms during adipogenesis have been previously reviewed in detail in [73,74,82,84,85]. Thus, in the context of the DOHaD, epigenetic remodeling during adipogenesis and their persistence (consistent with modified gene expression levels) may act as a “memory” for the perinatal environment experienced sensitizing offspring to obesity and its associated metabolic diseases later in life.

### 3.4. Formation of the Progenitor Cell Reservoir during Fetal Development

Adipose tissue can primarily expand in two ways: hypertrophy, which is the enlargement of existing adipocytes, and hyperplasia, which entails the formation of new adipocytes from preadipocyte precursors. Although the number of adipocytes in a specific depot is mainly established early in life and remains relatively stable through adulthood, differentiated adipocytes can significantly increase in size. However, recent studies in rodents have revealed that, during extended periods of excess calorie intake, new adipocytes can develop from preadipocytes, contributing to the expansion of adipose tissue [64,86,87,88,89]. These adult preadipocytes are typically fibroblast-like and often found near blood vessels [90,91,92]. The balance between hypertrophic expansion and adipogenesis in an individual significantly influences their metabolic health [68,93,94,95]. The establishment of APCs and adipocyte populations in adipose tissue occurs during early development, making the perinatal phase a crucial period that determines the trajectory of rapid fat accumulation during the neonatal and pubertal stages of development [9,18,68,96]. This perinatal phase programs the setpoint for adiposity in the long term [97].

### 3.5. Developmental Origin

Growing evidence indicates that fetal and adult adipogenesis are governed by distinct precursor cell populations and transcriptional regulatory mechanisms. Thus, distinct cell compartments may control adipose tissue development and homeostasis. Two distinct phases of adipocyte generation have been identified, originating from two independent APC sub-populations. One becomes active during the perinatal period, contributing to the development of adipose tissue, while the other is activated in adulthood for adipocyte turnover and the generation of new adipocytes if necessary. Despite both developmental and adult APCs being specified during the developmental period and expressing PPARγ, they exhibit distinct microanatomical, functional, morphogenetic, and molecular profiles [92].

In terms of cell population, some studies suggest that adult APCs arise from a specialized cell cluster present in mouse embryos at E10.5. This cluster is characterized by the expression of *Pparγ* and *Dlk1* genes [92,98]. These cells, located in the dorsal mesenteric region, actively contribute to the formation of adipocytes in adulthood and can persist throughout an individual’s life. However, they do not participate in the embryonic development of adipose tissue. These cells migrate to adipose tissue depots through the vascular system [92,98]. Other in vivo studies have shown that during fetal development of inguinal WAT, proliferating CD24+ APCs express *Plin1* and *Adipoq* [99], which are typically associated with mature adipocytes in adults [100]. These CD24+ PLIN1+ ADIPOQ+ APCs reside as clusters and are postnatally distributed along growing adipose vasculature (i.e., mural cell phenotype), in contrast to the adult preadipocytes present in the stromal vascular fraction [99].

At the molecular level, the role of some transcriptional factors is different depending on whether they govern fetal and adult adipogenesis. Thus, it has been observed that C/EBPα, a pro-adipogenic transcription factor, is not necessary for the maturation of APCs in inguinal and perigonadal WAT during postnatal development but plays a critical role in adult adipogenesis [101]. Likewise, the PI3K-AKT2 pathway, which is required for obesity-induced adipogenesis, does not participate in developmental WAT growth [87]. Recent findings also suggest that ZFP423, responsible for activating the master regulator of adipogenesis, PPARγ, plays a crucial role in the terminal differentiation of subcutaneous white adipocytes during fetal adipose tissue development, whereas it is mainly involved in maintaining the fate of white adipocytes in adult mice [102,103,104]. These studies reinforce the notion that developmental and adult APCs might be distinct populations, warranting separate investigation and characterization.

## 4. Determinants of Adipose Tissue Expansion during Health and Disease

### 4.1. Adipose Tissue Cellularity

The developmental and metabolic decisions shaping adipocyte cell number are set early in development. Therefore, disrupted formation of APCs and aberrant perinatal adipogenesis may permanently impact the adipocyte hyperplastic capacity, replacement, and overall cellularity throughout life. However, there are considerable inter-individual variations in the number of adipocytes, and the factors that determine this are yet unknown. In adulthood, adipose tissue size typically remains relatively stable under normal conditions but is highly responsive to dietary challenges, particularly high-calorie diets. Therefore, the total number and size of adipocytes largely remain unchanged after adolescence [105,106,107], with the replacement of adipocytes that have been turned over by newly formed adipocytes [68,86,108,109,110]. In young adult mice, it has been observed that approximately 1% to 1.5% of APCs undergo replication each day [111]. Additionally, the rate of adipogenesis in these mice has been estimated to range from 10% to 15% per month [86,110]. In humans, metabolic labeling studies using deuterium have indicated that adipocytes have a half-life of around 6 months, which translates to a turnover rate of approximately 60% per year [112]. However, retrospective studies utilizing radiocarbon (^14^C) birth dating of cells have indicated a considerably lower annual turnover rate for adipocytes, estimated at approximately 10% [68]. Nonetheless, it is worth noting that this turnover rate tends to gradually decrease with age due to the impaired regenerative capacity of APCs and the aging of the microenvironment [86,96].

In response to an excess of calories, mature adipocytes exhibit remarkable hypertrophic potential, capable of enlarging to several hundred micrometers in diameter [113]. Additionally, the expansion of fat mass can also occur through hyperplasia [64,86,87,88,114,115,116]. Importantly, the capacity to produce new adipocytes from APCs varies across different adipogenic sites (i.e., visceral vs. subcutaneous), and these processes are subject to distinct regulatory mechanisms with diverse outcomes. Studies in rodents suggest that female mice present more APCs in WAT compared to males [64,87,88]. For example, the increase in visceral WAT in males involves a combination of adipocyte hyperplasia and hypertrophy in both mice [64,88,103,117] and humans [118]. In male mice, subcutaneous WAT does not significantly undergo hyperplasia in response to obesogenic stimuli, possibly due to the microenvironmental conditions that suppress the APCs’ potential to undergo adipogenesis [64,87,88]. By contrast, female mice under HFD exhibit APC hyperplasia (probably mediated by estrogen) in both visceral and subcutaneous WAT [88]. Although hyperplasia is influenced by sex hormones in various WAT depots, the precise mechanism by which sex hormones determine adipocyte hyperplasia or hypertrophy is still not fully known [88]. Overall, these data underscore the existence of depot and sex-selective mechanisms governing adipose tissue expansion.

### 4.2. Adipose Tissue Expansion and Metabolic Diseases

Recent studies suggest that both the quality and quantity of adipose tissue play significant roles in maintaining metabolic health. It is well accepted that the accumulation of hypertrophic visceral WAT in males is detrimental to metabolic health, while hyperplastic subcutaneous WAT in females may be protective [88]. Thus, the impairment of the expandability of WAT (hypertrophy vs. hyperplasia) is considered to be one of the key factors in the development of obesity and related metabolic diseases later in life.

On the one hand, the adipose tissue expandability hypothesis posits that when subcutaneous WAT fails to adequately expand in response to energy stressors, it can lead to ectopic lipid accumulation in other organs (including skeletal muscle and liver), resulting in insulin resistance and type 2 diabetes [13]. In general, adipocyte hypertrophy is associated with a lower number of fat cells and insulin resistance, regardless of sex and body fat levels, whereas adipocyte hyperplasia is associated with better insulin sensitivity [93,95,119]. Thus, the disturbance of adipogenesis is associated with the risk of development of metabolic disorders [120,121], and hyperplastic obesity might have a protective role against such metabolic disturbances [122]. However, some studies indicate that people with insulin resistance or T2D have a higher proportion of small adipocytes, suggesting hyperplasia, compared to healthy individuals. Although there is some debate regarding the role of impaired adipogenesis in causing obesity-related problems [69], data suggest that difficulties in expanding small adipocytes are linked to insulin resistance [123,124,125,126] and that adipocyte number has significant implications for obesity management and prevention.

On the other hand, the adipocyte number hypothesis suggests that obesity in infancy results from an excessive increase in the number of adipocytes, leading to a higher adipocyte count throughout life and making individuals more susceptible to obesity at all ages [127,128]. This hypothesis is based on cross-sectional studies involving individuals without and with obesity [96,129] and validated by animal studies [105,130,131,132]. It aligns with the idea that infants with obesity are highly likely to suffer from it during adulthood [133]. Specifically, accelerated infant growth is linked to a higher risk of developing chronic non-communicable diseases, including obesity, T2D, and cardiovascular disease [134,135,136,137]. Rapid growth during infancy is primarily driven by overfeeding and excessive calorie intake [138,139].

**Figure 1:**
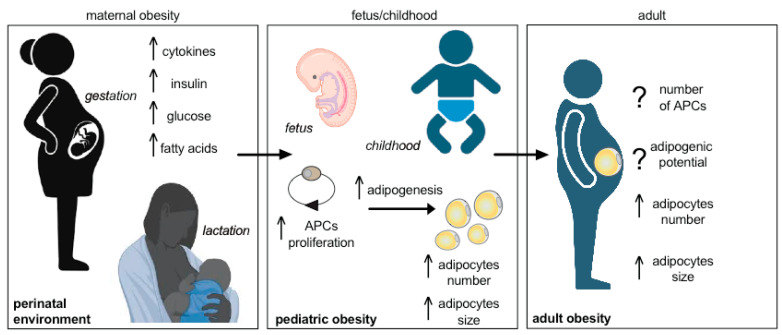
Perinatal environment plays a crucial role in adipogenesis, shaping both early and adult adipose tissue plasticity. Early-life obesity encourages the formation of fat cells (i.e., higher adipose precursor cells (APCs) proliferation) and programs the expansion of adipose tissue. It may result in exacerbated adipogenesis (i.e., higher hypertrophy and/or hyperplasia) throughout life. On the one hand, these processes may raise the body’s adiposity level, contributing to the onset of childhood obesity that may persist in adulthood. On the other hand, the APCs may deplete prematurely, leading to impaired adipose tissue plasticity and its associated metabolic diseases later in life.

Despite differences in developmental timing, rodents and humans share several mechanisms in WAT expansion. Rodent models have been mainly used to investigate the effects of maternal obesity on WAT development and function and to decipher underlying programming mechanisms. Accelerated neonatal growth can be induced in mice and rats by reducing litter size at birth or overfeeding mothers, leading to rapid weight gain and later metabolic issues like obesity, insulin resistance, and impaired glucose tolerance [140]. These long-term metabolic diseases can be attributed to alterations in the central nervous system, often resulting in increased energy intake or in peripheral tissues, especially adipose tissue, leading to increased lipid storage [9,38,39,141]. In particular, when discussing accelerated perinatal growth, we are referring to the swift accumulation of fat in perinatal WAT depots. This happens because the intrauterine environment and breast milk stimulate the proliferation of APCs, causing more stem cells to become adipocytes or undergo terminal differentiation. It has been recently shown that the number of embryonic APCs alone is decisive for the WAT mass in adult animals [142] (Figure 1). Maternal obesity has been linked to an acceleration in neonatal growth, an enhanced process of adipogenic differentiation within fetal adipose tissue, and an augmentation of central adiposity in offspring at the time of weaning [143,144,145,146]. Notably, these alterations in early-life adiposity can have enduring effects into adulthood (Figure 1). This phenomenon has been observed in various model species, including rodents, sheep, and non-human primates, which have shown an increased predisposition to obesity in the offspring of obese mothers.

In rat models, it has been consistently observed that male offspring of obese dams exhibit adipocyte hypertrophy compared to their counterparts from non-obese mothers [144,147,148,149]. However, the data regarding adipocyte hyperplasia is somewhat conflicting, with some studies suggesting an increase in adipocyte hyperplasia [148] while others indicate impairment [144]. Additionally, while both male and female offspring of obese mothers exhibit increased WAT mass, the mechanisms underlying this expansion seem to be sexually dimorphic. In the case of rat models of maternal obesity, an increase in adipocyte cell size has been observed in males, but this increase is not as pronounced in female offspring [148,149]. However, mechanisms underlying these observed differences between obesity-prone males and females remain elusive. In sheep models, fetuses of obese ewes develop increased fat depots due, at least in part, to adipocyte hypertrophy, particularly during late gestation when compared to fetuses of lean mothers [150,151,152,153] (Figure 1).

### 4.3. Link between Accelerated Perinatal Growth and Dysfunctional WAT in Offspring

The underlying mechanisms linking accelerated perinatal growth and dysfunctional WAT later in life remain elusive. Recent data suggest that epigenetic modifications could play a role in shaping WAT plasticity [9,154]. These negative effects on WAT development due to perinatal suboptimal nutrition may occur during critical periods, such as gestation and lactation, which establish the pool of APCs. During these phases, adipogenesis is highly active, and APCs are particularly sensitive to epigenetic changes. Fluctuations in energy and metabolic factors within cells, as well as alterations in hormonal signals in their microenvironment, can influence the development of APCs through epigenetic modifications that lead to changes in gene expression. Hence, the alteration of epigenetic marks on APCs during these critical developmental periods might underlie the long-term consequences of inadequate nutrient intake. These epigenetic marks can persist throughout an individual’s life and even be transmitted to future generations, potentially contributing to the transgenerational inheritance of obesity through epigenetic mechanisms. The concept of ongoing modification of early-life epigenetic imprints or memories during adulthood is supported by several studies [9,154].

Several studies have previously shown that both maternal obesity and accelerated growth during lactation can influence the plasticity of offspring’s WAT in obesity-prone rodents via transcriptional alterations in a sex- and depot-specific manner [9,149,154,155]. Recently, using a rat model of maternal diet-induced obesity, our group has reported data supporting the role of maternal obesity in the developmental programming of WAT through epigenetic mechanisms. Maternal obesity has distinct effects on the epigenetic remodeling of the promoter and enhancer sequences linked to higher leptin and lower *Pparγ* gene expression in both visceral and subcutaneous fat pads during development. It was also showed that epigenetic marks associated with persistent increased leptin and decreased *Pparγ* mRNA levels (i.e., methylation, hydroxymethylation, and histone post-translational modifications) are retained in a depot-specific manner into adulthood [156,157]. In line with these findings, using a mouse model, a recent study demonstrated that maternal obesity attenuates PPARγ nuclear migration in APCs early in gestation, impairing offspring organogenesis and adipogenesis by increasing preadipocyte proliferation in parallel with precocious lipid accumulation in subcutaneous fat pads [158]. During lactation, active epigenomic remodeling has been observed in the stearoyl-CoA desaturase1 gene (i.e., reduced DNA methylation in Scd1 promoter surrounding a PPARγ-binding region), a key enzyme of lipogenesis in visceral fat pads in obesity-prone male offspring [159]. Consistent with these findings, using a rat model of maternal diet-induced obesity, an increase in *Zfp423* mRNA expression levels and reduced DNA methylation in the promoter of stromal vascular cells isolated from visceral fat pads of obesity-prone adult offspring has been reported. These transcriptional and epigenetic modifications were linked to enhanced adipocyte differentiation and accelerated adipogenesis [160]. Another study, using a mouse model of maternal obesity, demonstrated that enhanced adipocyte progenitor cell differentiation and adipogenesis were associated with increased *Zfp423* gene expression through persistent epigenetic modifications (i.e., DNA hypomethylation in the promoter) from the fetal period to adulthood [161]. However, exacerbated adipogenesis during perinatal development impairs the proliferation of adipose progenitor cells and WAT plasticity in adulthood [144].

Such a failure in progenitor self-renewal might result in the exhaustion of the pool, as observed in adipose lineage-tracing experiments after prolonged rosiglitazone administration, a strong PPARγ agonist that promotes adipogenesis [114]. These findings shed light on a mechanism for the limited plasticity of WAT in the offspring of obese mothers, suggesting that the APC pool may be predisposed to exhaustion. If future studies confirm this phenomenon, it could emerge as a significant mechanism linking maternal obesity to premature aging and functional decline of WAT. In accordance with this observation of reduced expansion capacity, the offspring’s WAT of obese mothers exhibits larger adipocytes, a characteristic linked to inflammatory responses [162]. This inflammatory state in WAT is a hallmark of obesity-induced metabolic dysfunction resulting in impaired glucose tolerance and insulin sensitivity [11]. In line with these findings, some studies suggest that loss of WAT hyperplastic potential is associated with enhanced susceptibility to insulin resistance [11,86].

**Figure 2:**
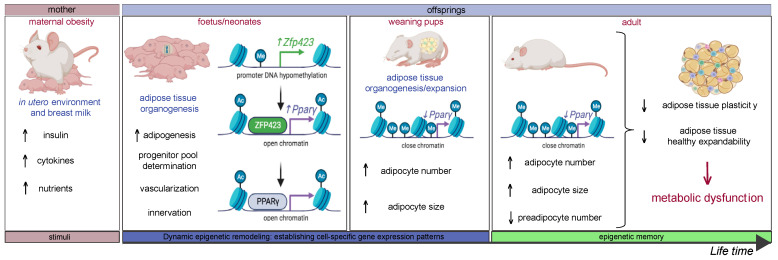
Epigenetic memory hypothesis: unraveling the mechanism connecting maternal obesity to adipose tissue dysfunction in offspring. Maternal obesity may create an unfavorable nutritional and hormonal milieu, influencing chromatin remodeling during perinatal adipogenesis. This intracellular remodeling may impact the gene-regulatory machinery and gene expression patterns, resulting in impaired adipocyte function. The epigenomic changes initiated in the perinatal period may persist into adulthood as an enduring epigenetic memory, contributing to a predisposition to obesity (i.e., higher adipocyte number and/or size, lower preadipocyte number, and impaired adipose tissue plasticity) and associated metabolic dysfunction later in life.

In summary, maternal obesity leads to persistent epigenetic changes in key genes that control the development of fat cells in the offspring’s WAT. This may disrupt the renewal of APCs during the expansion of WAT, hindering its ability to undergo further expansion. This process may result in hypertrophy of adipocytes, causing hypoxia and inflammation. These findings shed light on a critical and innovative mechanism connecting maternal obesity to the inflexibility of WAT and metabolic issues in offspring. It also introduces a new avenue for exploring developmental programming by examining epigenetic changes in APCs (Figure 2).

## 5. Conclusions

Early-life programming is becoming an established concept which states that the environment during early development affects health and disease in adulthood. Based on the DOHaD hypothesis, it will be increasingly important to better understand the involvement of epigenetic mechanisms in the programming of offspring’s APCs from obese dams. Thus, a better knowledge of the epigenomic changes that occur during the processes governing adipogenesis and adipogenic program in response to maternal obesity could be the basis for the development of therapeutic approaches as reprogramming strategies (i.e., nutritional interventions, pharmacological modifications of the epigenome) to reverse these programming effects. 

## Data Availability

Not applicable.

## Abbreviations

APC	adipose progenitor cells
BAT	brown adipose tissue
BMI	body mass index
DOHaD	Developmental Origin of Health and Disease
GDM	gestational diabetes mellitus
T2D	type 2 diabetes
WAT	white adipose tissue

## References

[1] Cunningham S.A., Hardy S.T., Jones R., Ng C., Kramer M.R., Narayan K.M.V. (2022). Changes in the Incidence of Childhood Obesity. Pediatrics.

[2] Karnik S., Kanekar A. (2012). Childhood Obesity: A Global Public Health Crisis. Int. J. Prev. Med..

[3] Boney C.M., Verma A., Tucker R., Vohr B.R. (2005). Metabolic syndrome in childhood: Association with birth weight, maternal obesity, and gestational diabetes mellitus. Pediatrics.

[4] Catalano P.M., Farrell K., Thomas A., Huston-Presley L., Mencin P., de Mouzon S.H., Amini S.B. (2009). Perinatal risk factors for childhood obesity and metabolic dysregulation. Am. J. Clin. Nutr..

[5] Ismail-Beigi F., Catalano P.M., Hanson R.W. (2006). Metabolic programming: Fetal origins of obesity and metabolic syndrome in the adult. Am. J. Physiol. Endocrinol. Metab..

[6] Oken E., Gillman M.W. (2003). Fetal origins of obesity. Obes. Res..

[7] Wu A.J., Oken E. (2023). Developmental Contributions to Obesity: Nutritional Exposures in the First Thousand Days. Gastroenterol. Clin. N. Am..

[8] Whitaker R.C. (2004). Predicting preschooler obesity at birth: The role of maternal obesity in early pregnancy. Pediatrics.

[9] Lecoutre S., Petrus P., Rydén M., Breton C. (2018). Transgenerational Epigenetic Mechanisms in Adipose Tissue Development. Trends Endocrinol. Metab..

[10] González-Muniesa P., Mártinez-González M.-A., Hu F.B., Després J.-P., Matsuzawa Y., Loos R.J.F., Moreno L.A., Bray G.A., Martinez J.A. (2017). Obesity. Nat. Rev. Dis. Prim..

[11] Lecoutre S., Lambert M., Drygalski K., Dugail I., Maqdasy S., Hautefeuille M., Clément K. (2022). Importance of the Microenvironment and Mechanosensing in Adipose Tissue Biology. Cells.

[12] Czech M.P. (2020). Mechanisms of insulin resistance related to white, beige, and brown adipocytes. Mol. Metab..

[13] Virtue S., Vidal-Puig A. (2010). Adipose tissue expandability, lipotoxicity and the Metabolic Syndrome—An allostatic perspective. Biochim. Biophys. Acta Mol. Cell Biol. Lipids.

[14] Pellegrinelli V., Carobbio S., Vidal-Puig A. (2016). Adipose tissue plasticity: How fat depots respond differently to pathophysiological cues. Diabetologia.

[15] Reilly S.M., Saltiel A.R. (2017). Adapting to obesity with adipose tissue inflammation. Nat. Rev. Endocrinol..

[16] Lumeng C.N., Saltiel A.R. (2011). Inflammatory links between obesity and metabolic disease. J. Clin. Investig..

[17] Han J., Lee J.-E., Jin J., Lim J.S., Oh N., Kim K., Chang S.-I., Shibuya M., Kim H., Koh G.Y. (2011). The spatiotemporal development of adipose tissue. Development.

[18] Holtrup B., Church C.D., Berry R., Colman L., Jeffery E., Bober J., Rodeheffer M.S. (2017). Puberty is an important developmental period for the establishment of adipose tissue mass and metabolic homeostasis. Adipocyte.

[19] Berry D.C., Stenesen D., Zeve D., Graff J.M. (2013). The developmental origins of adipose tissue. Development.

[20] Barker D.J.P. (2004). Developmental origins of adult health and disease. J. Epidemiol. Community Health.

[21] Painter R.C., Osmond C., Gluckman P., Hanson M., Phillips D.I.W., Roseboom T.J. (2008). Transgenerational effects of prenatal exposure to the Dutch famine on neonatal adiposity and health in later life. BJOG.

[22] Li C., Lumey L.H. (2017). Exposure to the Chinese famine of 1959-61 in early life and long-term health conditions: A systematic review and meta-analysis. Int. J. Epidemiol..

[23] Safi-Stibler S., Gabory A. (2020). Epigenetics and the Developmental Origins of Health and Disease: Parental environment signalling to the epigenome, critical time windows and sculpting the adult phenotype. Semin. Cell Dev. Biol..

[24] Godfrey K.M., Gluckman P.D., Hanson M.A. (2010). Developmental origins of metabolic disease: Life course and intergenerational perspectives. Trends Endocrinol. Metab..

[25] Lumey L.H., Stein A.D., Susser E. (2011). Prenatal Famine and Adult Health. Annu. Rev. Public Health.

[26] Langley-Evans S.C., Pearce J., Ellis S. (2022). Overweight, obesity and excessive weight gain in pregnancy as risk factors for adverse pregnancy outcomes: A narrative review. J. Hum. Nutr. Diet..

[27] Leddy M.A., Power M.L., Schulkin J. (2008). The impact of maternal obesity on maternal and fetal health. Rev. Obstet. Gynecol..

[28] Gilbert J.A.L. (1949). The association of maternal obesity, large babies, and diabetes. Br. Med. J..

[29] Pedersen J., Bojsen-Møller B., Poulsen H. (1954). Blood sugar in newborn infants of diabetic mothers. Acta Endocrinol. (Copenh).

[30] Catalano P.M., McIntyre H.D., Cruickshank J.K., McCance D.R., Dyer A.R., Metzger B.E., Lowe L.P., Trimble E.R., Coustan D.R., Hadden D.R. (2012). The hyperglycemia and adverse pregnancy outcome study: Associations of GDM and obesity with pregnancy outcomes. Diabetes Care.

[31] Reynolds R.M., Allan K.M., Raja E.A., Bhattacharya S., McNeill G., Hannaford P.C., Sarwar N., Lee A.J., Bhattacharya S., Norman J.E. (2013). Maternal obesity during pregnancy and premature mortality from cardiovascular event in adult offspring: Follow-up of 1 323 275 person years. BMJ.

[32] Drake A.J., Reynolds R.M. (2010). Impact of maternal obesity on offspring obesity and cardiometabolic disease risk. Reproduction.

[33] Kral J.G., Biron S., Simard S., Hould F.S., Lebel S., Marceau S., Marceau P. (2006). Large maternal weight loss from obesity surgery prevents transmission of obesity to children who were followed for 2 to 18 years. Pediatrics.

[34] Damm P., Houshmand-Oeregaard A., Kelstrup L., Lauenborg J., Mathiesen E.R., Clausen T.D. (2016). Gestational diabetes mellitus and long-term consequences for mother and offspring: A view from Denmark. Diabetologia.

[35] Modi N., Murgasova D., Ruager-Martin R., Thomas E.L., Hyde M.J., Gale C., Santhakumaran S., Doré C.J., Alavi A., Bell J.D. (2011). The influence of maternal body mass index on infant adiposity and hepatic lipid content. Pediatr. Res..

[36] Guenard F., Deshaies Y., Cianflone K., Kral J.G., Marceau P., Vohl M.-C. (2013). Differential methylation in glucoregulatory genes of offspring born before *vs*. after maternal gastrointestinal bypass surgery. Proc. Natl. Acad. Sci. USA.

[37] Desai M., Jellyman J.K., Ross M.G. (2015). Epigenomics, gestational programming and risk of metabolic syndrome. Int. J. Obes. (Lond.).

[38] Steculorum S.M., Bouret S.G. (2011). Maternal diabetes compromises the organization of hypothalamic feeding circuits and impairs leptin sensitivity in offspring. Endocrinology.

[39] Vogt M.C., Paeger L., Hess S., Steculorum S.M., Awazawa M., Hampel B., Neupert S., Nicholls H.T., Mauer J., Hausen A.C. (2014). Neonatal insulin action impairs hypothalamic neurocircuit formation in response to maternal high-fat feeding. Cell.

[40] Rolls B.A., Gurr M.I., Van Duijvenvoorde P.M., Rolls B.J., Rowe E.A. (1986). Lactation in lean and obese rats: Effect of cafeteria feeding and of dietary obesity on milk composition. Physiol. Behav..

[41] Marousez L., Lesage J., Eberlé D. (2019). Epigenetics: Linking Early Postnatal Nutrition to Obesity Programming?. Nutrients.

[42] Bautista C.J., Montaño S., Ramirez V., Morales A., Nathanielsz P.W., Bobadilla N.A., Zambrano E. (2016). Changes in milk composition in obese rats consuming a high-fat diet. Br. J. Nutr..

[43] Castillo P., Kuda O., Kopecky J., Pomar C.A., Palou A., Palou M., Picó C. (2022). Reverting to a Healthy Diet during Lactation Normalizes Maternal Milk Lipid Content of Diet-Induced Obese Rats and Prevents Early Alterations in the Plasma Lipidome of the Offspring. Mol. Nutr. Food Res..

[44] Pomar C.A., Kuda O., Kopecky J., Rombaldova M., Castro H., Picó C., Sánchez J., Palou A. (2020). Maternal diet, rather than obesity itself, has a main influence on milk triacylglycerol profile in dietary obese rats. Biochim. Biophys. Acta. Mol. Cell Biol. Lipids.

[45] Donato J. (2023). Programming of metabolism by adipokines during development. Nat. Rev. Endocrinol..

[46] Uysal F.K., Önal E.E., Aral Y.Z., Adam B., Dilmen U., Ardiçolu Y. (2002). Breast milk leptin: Its relationship to maternal and infant adiposity. Clin. Nutr..

[47] Houseknecht K.L., McGuire M.K., Portocarrero C.P., McGuire M.A., Beerman K. (1997). Leptin is present in human milk and is related to maternal plasma leptin concentration and adiposity. Biochem. Biophys. Res. Commun..

[48] Schuster S., Hechler C., Gebauer C., Kiess W., Kratzsch J. (2011). Leptin in maternal serum and breast milk: Association with infants’ body weight gain in a longitudinal study over 6 months of lactation. Pediatr. Res..

[49] Christensen S.H., Lewis J.I., Larnkjær A., Frøkiær H., Allen L.H., Mølgaard C., Michaelsen K.F. (2022). Associations between maternal adiposity and appetite-regulating hormones in human milk are mediated through maternal circulating concentrations and might affect infant outcomes. Front. Nutr..

[50] Mohamad M., Loy S.L., Lim P.Y., Wang Y., Soo K.L., Mohamed H.J.J. (2018). Maternal Serum and Breast Milk Adiponectin: The Association with Infant Adiposity Development. Int. J. Environ. Res. Public Health.

[51] Buonfiglio D.C., Ramos-Lobo A.M., Freitas V.M., Zampieri T.T., Nagaishi V.S., Magalhães M., Cipolla-Neto J., Cella N., Donato J. (2016). Obesity impairs lactation performance in mice by inducing prolactin resistance. Sci. Rep..

[52] Pena-Leon V., Folgueira C., Barja-Fernández S., Pérez-Lois R., Da Silva Lima N., Martin M., Heras V., Martinez-Martinez S., Valero P., Iglesias C. (2022). Prolonged breastfeeding protects from obesity by hypothalamic action of hepatic FGF21. Nat. Metab..

[53] Da Silva Lima N., Gaspar De Moura E., Cottini Fonseca Passos M., Firmino Nogueira Neto J., Reis A.M., De Oliveira E., Lisboa P.C. (2011). Early weaning causes undernutrition for a short period and programmes some metabolic syndrome components and leptin resistance in adult rat offspring. Br. J. Nutr..

[54] Lukaszewski M.-A., Eberlé D., Vieau D., Breton C. (2013). Nutritional manipulations in the perinatal period program adipose tissue in offspring. Am. J. Physiol. Endocrinol. Metab..

[55] Rosen E.D., Spiegelman B.M. (2014). What we talk about when we talk about fat. Cell.

[56] Auger C., Kajimura S. (2023). Adipose Tissue Remodeling in Pathophysiology. Annu. Rev. Pathol..

[57] Chouchani E.T., Kajimura S. (2019). Metabolic adaptation and maladaptation in adipose tissue. Nat. Metab..

[58] Morigny P., Boucher J., Arner P., Langin D. (2021). Lipid and glucose metabolism in white adipocytes: Pathways, dysfunction and therapeutics. Nat. Rev. Endocrinol..

[59] Marcelin G., Gautier E.L., Clement K. (2022). Adipose Tissue Fibrosis in Obesity: Etiology and Challenges. Annu. Rev. Physiol..

[60] Toubal A., Treuter E., Clément K., Venteclef N. (2013). Genomic and epigenomic regulation of adipose tissue inflammation in obesity. Trends Endocrinol. Metab..

[61] Ailhaud G., Grimaldi P., Négrel R. (1992). Cellular and molecular aspects of adipose tissue development. Annu. Rev. Nutr..

[62] Berry D.C., Jiang Y., Graff J.M. (2016). Emerging roles of adipose progenitor cells in tissue development, homeostasis, expansion and thermogenesis. Trends Endocrinol. Metab..

[63] Birsoy K., Berry R., Wang T., Ceyhan O., Tavazoie S., Friedman J.M., Rodeheffer M.S. (2011). Analysis of gene networks in white adipose tissue development reveals a role for ETS2 in adipogenesis. Development.

[64] Wang Q.A., Tao C., Gupta R.K., Scherer P.E. (2013). Tracking adipogenesis during white adipose tissue development, expansion and regeneration. Nat. Med..

[65] Poissonnet C.M., Burdi A.R., Garn S.M. (1984). The chronology of adipose tissue appearance and distribution in the human fetus. Early Hum. Dev..

[66] Crandall D.L., Hausman G.J., Kral J.G. (1997). A review of the microcirculation of adipose tissue: Anatomic, metabolic, and angiogenic perspectives. Microcirculation.

[67] Knittle J.L., Timmers K., Ginsberg-Fellner F., Brown R.E., Katz D.P. (1979). The growth of adipose tissue in children and adolescents. Cross-sectional and longitudinal studies of adipose cell number and size. J. Clin. Investig..

[68] Spalding K.L., Arner E., Westermark P.O., Bernard S., Buchholz B.A., Bergmann O., Blomqvist L., Hoffstedt J., Näslund E., Britton T. (2008). Dynamics of fat cell turnover in humans. Nature.

[69] White U., Ravussin E. (2019). Dynamics of adipose tissue turnover in human metabolic health and disease. Diabetologia.

[70] Ryan T.J., Curri S.B. (1989). The development of adipose tissue and its relationship to the vascular system. Clin. Dermatol..

[71] Cannon B., Nedergaard J. (2004). Brown adipose tissue: Function and physiological significance. Physiol. Rev..

[72] Ghaben A.L., Scherer P.E. (2019). Adipogenesis and metabolic health. Nat. Rev. Mol. Cell Biol..

[73] Cristancho A.G., Lazar M.A. (2011). Forming functional fat: A growing understanding of adipocyte differentiation. Nat. Rev. Mol. Cell Biol..

[74] Siersbæk R., Nielsen R., Mandrup S. (2012). Transcriptional networks and chromatin remodeling controlling adipogenesis. Trends Endocrinol. Metab..

[75] Ross S.E., Hemati N., Longo K.A., Bennett C.N., Lucas P.C., Erickson R.L., MacDougald O.A. (2000). Inhibition of adipogenesis by Wnt signaling. Science.

[76] Huang H., Song T.J., Li X., Hu L., He Q., Liu M., Lane M.D., Tang Q.Q. (2009). BMP signaling pathway is required for commitment of C3H10T1/2 pluripotent stem cells to the adipocyte lineage. Proc. Natl. Acad. Sci. USA.

[77] Hammarstedt A., Hedjazifar S., Jenndahl L., Gogg S., Grünberg J., Gustafson B., Klimcakova E., Stich V., Langin D., Laakso M. (2013). WISP2 regulates preadipocyte commitment and PPARγ activation by BMP4. Proc. Natl. Acad. Sci. USA.

[78] Lefterova M.I., Zhang Y., Steger D.J., Schupp M., Schug J., Cristancho A., Feng D., Zhuo D., Stoeckert C.J., Liu X.S. (2008). PPAR gamma and C/EBP factors orchestrate adipocyte biology *via* adjacent binding on a genome-wide scale. Genes Dev..

[79] Lefterova M.I., Haakonsson A.K., Lazar M.A., Mandrup S. (2014). PPAR gamma and the global map of adipogenesis and beyond. Trends Endocrinol. Metab..

[80] Tontonoz P., Hu E., Spiegelman B.M. (1994). Stimulation of adipogenesis in fibroblasts by PPAR gamma 2, a lipid-activated transcription factor. Cell.

[81] Mikkelsen T.S., Xu Z., Zhang X., Wang L., Gimble J.M., Lander E.S., Rosen E.D. (2010). Comparative epigenomic analysis of murine and human adipogenesis. Cell.

[82] Steger D.J., Grant G.R., Schupp M., Tomaru T., Lefterova M.I., Schug J., Manduchi E., Stoeckert C.J., Lazar M.A. (2010). Propagation of adipogenic signals through an epigenomic transition state. Genes Dev..

[83] Siersbaek R., Nielsen R., John S., Sung M.-H., Baek S., Loft A., Hager G.L., Mandrup S. (2011). Extensive chromatin remodelling and establishment of transcription factor ‘hotspots’ during early adipogenesis. EMBO J..

[84] Inagaki T., Sakai J., Kajimura S. (2016). Transcriptional and epigenetic control of brown and beige adipose cell fate and function. Nat. Rev. Mol. Cell Biol..

[85] Öst A., Pospisilik J.A. (2015). Epigenetic modulation of metabolic decisions. Curr. Opin. Cell Biol..

[86] Kim S.M., Lun M., Wang M., Senyo S.E., Guillermier C., Patwari P., Steinhauser M.L. (2014). Loss of White Adipose Hyperplastic Potential Is Associated with Enhanced Susceptibility to Insulin Resistance. Cell Metab..

[87] Jeffery E., Church C.D., Holtrup B., Colman L., Rodeheffer M.S. (2015). Rapid depot-specific activation of adipocyte precursor cells at the onset of obesity. Nat. Cell Biol..

[88] Jeffery E., Wing A., Holtrup B., Sebo Z., Kaplan J.L., Saavedra-Peña R., Church C.D., Colman L., Berry R., Rodeheffer M.S. (2016). The Adipose Tissue Microenvironment Regulates Depot-Specific Adipogenesis in Obesity. Cell Metab..

[89] Tchoukalova Y.D., Votruba S.B., Tchkonia T., Giorgadze N., Kirkland J.L., Jensen M.D. (2010). Regional differences in cellular mechanisms of adipose tissue gain with overfeeding. Proc. Natl. Acad. Sci. USA.

[90] Tang Q.Q., Lane M.D. (2012). Adipogenesis: From stem cell to adipocyte. Annu. Rev. Biochem..

[91] Tang W., Zeve D., Suh J.M., Bosnakovski D., Kyba M., Hammer R.E., Tallquist M.D., Graff J.M. (2008). White Fat Progenitor Cells Reside in the Adipose Vasculature. Science.

[92] Jiang Y., Berry D.C., Tang W., Graff J.M. (2014). Independent Stem Cell Lineages Regulate Adipose Organogenesis and Adipose Homeostasis. Cell Rep..

[93] Hoffstedt J., Arner E., Wahrenberg H., Andersson D.P., Qvisth V., Löfgren P., Rydén M., Thörne A., Wirén M., Palmér M. (2010). Regional impact of adipose tissue morphology on the metabolic profile in morbid obesity. Diabetologia.

[94] Acosta J.R., Douagi I., Andersson D.P., Bäckdahl J., Rydén M., Arner P., Laurencikiene J. (2016). Increased fat cell size: A major phenotype of subcutaneous white adipose tissue in non-obese individuals with type 2 diabetes. Diabetologia.

[95] Arner E., Westermark P.O., Spalding K.L., Britton T., Rydén M., Frisén J., Bernard S., Arner P. (2010). Adipocyte turnover: Relevance to human adipose tissue morphology. Diabetes.

[96] Guillermier C., Fazeli P.K., Kim S., Lun M., Zuflacht J.P., Milian J., Lee H., Francois-Saint-Cyr H., Horreard F., Larson D. (2017). Imaging mass spectrometry demonstrates age-related decline in human adipose plasticity. JCI Insight.

[97] Lecoutre S., Breton C. (2014). The cellularity of offspring’s adipose tissue is programmed by maternal nutritional manipulations. Adipocyte.

[98] Hudak C.S., Gulyaeva O., Wang Y., Park S., Lee L., Kang C., Sul H.S. (2014). Pref-1 Marks Very Early Mesenchymal Precursors Required for Adipose Tissue Development and Expansion. Cell Rep..

[99] Hong K.Y., Bae H., Park I., Park D.Y., Kim K.H., Kubota Y., Cho E.S., Kim H., Adams R.H., Yoo O.J. (2015). Perilipin+ embryonic preadipocytes actively proliferate along growing vasculatures for adipose expansion. Development.

[100] Scherer P.E., Williams S., Fogliano M., Baldini G., Lodish H.F. (1995). A novel serum protein similar to C1q, produced exclusively in adipocytes. J. Biol. Chem..

[101] Wang Q.A., Tao C., Jiang L., Shao M., Ye R., Zhu Y., Gordillo R., Ali A., Lian Y., Holland W.L. (2015). Distinct regulatory mechanisms governing embryonic versus adult adipocyte maturation. Nat. Cell Biol..

[102] Gupta R.K., Arany Z., Seale P., Mepani R.J., Ye L., Conroe H.M., Roby Y.A., Kulaga H., Reed R.R., Spiegelman B.M. (2010). Transcriptional control of preadipocyte determination by Zfp423. Nature.

[103] Vishvanath L., MacPherson K.A., Hepler C., Wang Q.A., Shao M., Spurgin S.B., Wang M.Y., Kusminski C.M., Morley T.S., Gupta R.K. (2015). Pdgfrβ+ Mural Preadipocytes Contribute to Adipocyte Hyperplasia Induced by High-Fat-Diet Feeding and Prolonged Cold Exposure in Adult Mice. Cell Metab..

[104] Shao M., Hepler C., Vishvanath L., MacPherson K.A., Busbuso N.C., Gupta R.K. (2017). Fetal development of subcutaneous white adipose tissue is dependent on Zfp423. Mol. Metab..

[105] Knittle J.L., Hirsch J. (1968). Effect of early nutrition on the development of rat epididymal fat pads: Cellularity and metabolism. J. Clin. Investig..

[106] Greenwood M.R., Hirsch J. (1974). Postnatal development of adipocyte cellularity in the normal rat. J. Lipid Res..

[107] Lemonnier D. (1972). Effect of age, sex, and sites on the cellularity of the adipose tissue in mice and rats rendered obese by a high-fat diet. J. Clin. Investig..

[108] Stiles J.W., Francendese A.A., Masoro E.J. (1975). Influence of age on size and number of fat cells in the epididymal depot. Am. J. Physiol..

[109] Hemmeryckx B., Loeckx D., Dresselaers T., Himmelreich U., Hoylaerts M.F., Roger Lijnen H. (2010). Age-associated adaptations in murine adipose tissues. Endocr. J..

[110] Rigamonti A., Brennand K., Lau F., Cowan C.A. (2011). Rapid Cellular Turnover in Adipose Tissue. PLoS ONE.

[111] Neese R.A., Misell L.M., Turner S., Chu A., Kim J., Cesar D., Hoh R., Antelo F., Strawford A., McCune J.M. (2002). Measurement in vivo of proliferation rates of slow turnover cells by 2H2O labeling of the deoxyribose moiety of DNA. Proc. Natl. Acad. Sci. USA.

[112] Strawford A., Antelo F., Christiansen M., Hellerstein M.K. (2004). Adipose tissue triglyceride turnover, de novo lipogenesis, and cell proliferation in humans measured with 2H_2_O. Am. J. Physiol.-Endocrinol. Metab..

[113] Hirsch J., Han P.W. (1969). Cellularity of rat adipose tissue: Effects of growth, starvation, and obesity. J. Lipid Res..

[114] Tang W., Zeve D., Seo J., Jo A.Y., Graff J.M. (2011). Thiazolidinediones regulate adipose lineage dynamics. Cell Metab..

[115] Faust I.M., Johnson P.R., Stern J.S., Hirsch J. (1978). Diet-induced adipocyte number increase in adult rats: A new model of obesity. Am. J. Physiol..

[116] Jo J., Gavrilova O., Pack S., Jou W., Mullen S., Sumner A.E., Cushman S.W., Periwal V. (2009). Hypertrophy and/or hyperplasia: Dynamics of adipose tissue growth. PLoS Comput. Biol..

[117] Stefkovich M., Traynor S., Cheng L., Merrick D., Seale P. (2021). Dpp4+ interstitial progenitor cells contribute to basal and high fat diet-induced adipogenesis. Mol. Metab..

[118] Arner P., Andersson D.P., Thörne A., Wirén M., Hoffstedt J., Näslund E., Thorell A., Rydén M. (2013). Variations in the size of the major omentum are primarily determined by fat cell number. J. Clin. Endocrinol. Metab..

[119] Arner P., Spalding K.L. (2010). Fat cell turnover in humans. Biochem. Biophys. Res. Commun..

[120] Weyer C., Foley J.E., Bogardus C., Tataranni P.A., Pratley R.E. (2000). Enlarged subcutaneous abdominal adipocyte size, but not obesity itself, predicts type II diabetes independent of insulin resistance. Diabetologia.

[121] Lessard J., Laforest S., Pelletier M., Leboeuf M., Blackburn L., Tchernof A. (2014). Low abdominal subcutaneous preadipocyte adipogenesis is associated with visceral obesity, visceral adipocyte hypertrophy, and a dysmetabolic state. Adipocyte.

[122] Kim J.-Y., van de Wall E., Laplante M., Azzara A., Trujillo M.E., Hofmann S.M., Schraw T., Durand J.L., Li H., Li G. (2007). Obesity-associated improvements in metabolic profile through expansion of adipose tissue. J. Clin. Investig..

[123] McLaughlin T., Lamendola C., Coghlan N., Liu T.C., Lerner K., Sherman A., Cushman S.W. (2014). Subcutaneous adipose cell size and distribution: Relationship to insulin resistance and body fat. Obesity (Silver Spring).

[124] McLaughlin T., Sherman A., Tsao P., Gonzalez O., Yee G., Lamendola C., Reaven G.M., Cushman S.W. (2007). Enhanced proportion of small adipose cells in insulin-resistant *vs* insulin-sensitive obese individuals implicates impaired adipogenesis. Diabetologia.

[125] Pasarica M., Tchoukalova Y.D., Heilbronn L.K., Fang X., Albu J.B., Kelley D.E., Smith S.R., Ravussin E. (2009). Differential effect of weight loss on adipocyte size subfractions in patients with type 2 diabetes. Obesity (Silver Spring).

[126] White U., Beyl R.A., Ravussin E. (2022). A higher proportion of small adipocytes is associated with increased visceral and ectopic lipid accumulation during weight gain in response to overfeeding in men. Int. J. Obes. (Lond.).

[127] Roche A.F. (1981). The Adipocyte-Number Hypothesis. Child Dev..

[128] Brook C.G.D., Lloyd J.K., Wolf O.H. (1972). Relation between age of onset of obesity and size and number of adipose cells. Br. Med. J..

[129] Salans L.B., Cushman S.W., Weismann R.E. (1973). Studies of Human Adipose Tissue adipose cell size and number in nonobese and obese patients. J. Clin. Investig..

[130] Enesco M., Leblond C.P. (1962). Increase in Cell Number as a Factor in the Growth of the Organs and Tissues of the Young Male Rat. Development.

[131] Winick M., Noble A. (1966). Cellular response in rats during malnutrition at various ages. J. Nutr..

[132] Winick M., Noble A. (1965). Quantitative changes in DNA, RNA, and protein during prenatal and postnatal growth in the rat. Dev. Biol..

[133] Wolff O.H., Lloyd J.K. (1973). Childhood obesity. Proc. Nutr. Soc..

[134] Stettler N., Zemel B.S., Kumanyika S., Stallings V.A. (2002). Infant weight gain and childhood overweight status in a multicenter, cohort study. Pediatrics.

[135] Gluckman P.D., Hanson M.A., Cooper C., Thornburg K.L. (2008). Effect of in utero and early-life conditions on adult health and disease. N. Engl. J. Med..

[136] Leunissen R.W.J., Stijnen T., Hokken-Koelega A.C.S. (2009). Influence of birth size on body composition in early adulthood: The programming factors for growth and metabolism (PROGRAM)-study. Clin. Endocrinol. (Oxf.).

[137] Harnois-Leblanc S., Van Hulst A., Henderson M. (2019). Persistence of Obesity from Early Childhood Onward. N. Engl. J. Med..

[138] Singhal A., Kennedy K., Lanigan J., Fewtrell M., Cole T.J., Stephenson T., Elias-Jones A., Weaver L.T., Ibhanesebhor S., MacDonald P.D. (2010). Nutrition in infancy and long-term risk of obesity: Evidence from 2 randomized controlled trials. Am. J. Clin. Nutr..

[139] Singhal A. (2010). Does weight gain in infancy influence the later risk of obesity?. J. Pediatr. Gastroenterol. Nutr..

[140] Ozanne S.E., Hales C.N. (2004). Lifespan: Catch-up growth and obesity in male mice. Nature.

[141] Plagemann A. (2005). Perinatal programming and functional teratogenesis: Impact on body weight regulation and obesity. Physiol. Behav..

[142] Hedbacker K., Lu Y.H., Dallner O., Li Z., Fayzikhodjaeva G., Birsoy K., Han C., Yang C., Friedman J.M. (2020). Limitation of adipose tissue by the number of embryonic progenitor cells. Elife.

[143] Samuelsson A.-M., Matthews P.A., Argenton M., Christie M.R., McConnell J.M., Jansen E.H.J.M., Piersma A.H., Ozanne S.E., Twinn D.F., Remacle C. (2008). Diet-induced obesity in female mice leads to offspring hyperphagia, adiposity, hypertension, and insulin resistance: A novel murine model of developmental programming. Hypertension.

[144] Liang X., Yang Q., Fu X., Rogers C.J., Wang B., Pan H., Zhu M.-J., Nathanielsz P.W., Du M. (2016). Maternal obesity epigenetically alters visceral fat progenitor cell properties in male offspring mice. J. Physiol..

[145] Shankar K., Harrell A., Liu X., Gilchrist J.M., Ronis M.J.J., Badger T.M. (2008). Maternal obesity at conception programs obesity in the offspring. Am. J. Physiol. Regul. Integr. Comp. Physiol..

[146] Dugail I., Quignard-Boulangé A., Dupuy F. (1986). Role of adipocyte precursors in the onset of obesity induced by overfeeding in suckling rats. J. Nutr..

[147] Zambrano E., Martínez-Samayoa P.M., Rodríguez-González G.L., Nathanielsz P.W. (2010). Dietary intervention prior to pregnancy reverses metabolic programming in male offspring of obese rats. J. Physiol..

[148] Lecoutre S., Deracinois B., Laborie C., Eberlé D., Guinez C., Panchenko P.E., Lesage J., Vieau D., Junien C., Gabory A. (2016). Depot- and sex-specific effects of maternal obesity in offspring’s adipose tissue. J. Endocrinol..

[149] Litzenburger T., Huber E.K., Dinger K., Wilke R., Vohlen C., Selle J., Kadah M., Persigehl T., Heneweer C., Dotsch J. (2020). Maternal high-fat diet induces long-term obesity with sex-dependent metabolic programming of adipocyte differentiation, hypertrophy and dysfunction in the offspring. Clin. Sci. (Lond.).

[150] Mühlhäusler B.S., Roberts C.T., Yuen B.S.J., Marrocco E., Budge H., Symonds M.E., McFarlane J.R., Kauter K.G., Stagg P., Pearse J.K. (2003). Determinants of fetal leptin synthesis, fat mass, and circulating leptin concentrations in well-nourished ewes in late pregnancy. Endocrinology.

[151] Muhlhausler B.S., Duffield J.A., McMillen I.C. (2007). Increased maternal nutrition stimulates peroxisome proliferator activated receptor-gamma, adiponectin, and leptin messenger ribonucleic acid expression in adipose tissue before birth. Endocrinology.

[152] Fensterseifer S.R., Austin K.J., Ford S.P., Alexander B.M. (2018). Effects of maternal obesity on maternal and fetal plasma concentrations of adiponectin and expression of adiponectin and its receptor genes in cotyledonary and adipose tissues at mid- and late-gestation in sheep. Anim. Reprod. Sci..

[153] Long N.M., Rule D.C., Zhu M.J., Nathanielsz P.W., Ford S.P. (2012). Maternal obesity upregulates fatty acid and glucose transporters and increases expression of enzymes mediating fatty acid biosynthesis in fetal adipose tissue depots. J. Anim. Sci..

[154] Lecoutre S., Kwok K.H.M., Petrus P., Lambert M., Breton C. (2019). Epigenetic programming of adipose tissue in the progeny of obese dams. Curr. Genom..

[155] Sellayah D., Thomas H., Lanham S.A., Cagampang F.R. (2019). Maternal Obesity During Pregnancy and Lactation Influences Offspring Obesogenic Adipogenesis but Not Developmental Adipogenesis in Mice. Nutrients.

[156] Lecoutre S., Pourpe C., Butruille L., Marousez L., Laborie C., Guinez C., Lesage J., Vieau D., Eeckhoute J., Gabory A. (2018). Reduced PPARγ2 expression in adipose tissue of male rat offspring from obese dams is associated with epigenetic modifications. FASEB J..

[157] Lecoutre S., Oger F., Pourpe C., Butruille L., Marousez L., Dickes-Coopman A., Laborie C., Guinez C., Lesage J., Vieau D. (2017). Maternal obesity programs increased leptin gene expression in rat male offspring *via* epigenetic modifications in a depot-specific manner. Mol. Metab..

[158] de Sousa É., Rodrigues A.C. (2023). Maternal obesity attenuates PPARγ nuclear migration impairing offspring adipogenesis. J. Mol. Endocrinol..

[159] Butruille L., Marousez L., Pourpe C., Oger F., Lecoutre S., Catheline D., Görs S., Metges C.C., Guinez C., Laborie C. (2019). Maternal high-fat diet during suckling programs visceral adiposity and epigenetic regulation of adipose tissue stearoyl-CoA desaturase-1 in offspring. Int. J. Obes..

[160] Borengasser S.J., Zhong Y., Kang P., Lindsey F., Ronis M.J.J., Badger T.M., Gomez-Acevedo H., Shankar K. (2013). Maternal obesity enhances white adipose tissue differentiation and alters genome-scale DNA methylation in male rat offspring. Endocrinology.

[161] Yang Q.-Y., Liang J.-F., Rogers C.J., Zhao J.-X., Zhu M.-J., Du M. (2013). Maternal obesity induces epigenetic modifications to facilitate Zfp423 expression and enhance adipogenic differentiation in fetal mice. Diabetes.

[162] Alfaradhi M.Z., Kusinski L.C., Fernandez-Twinn D.S., Pantaleão L.C., Carr S.K., Ferland-McCollough D., Yeo G.S.H., Bushell M., Ozanne S.E. (2016). Maternal obesity in pregnancy developmentally programs adipose tissue inflammation in young, lean male mice offspring. Endocrinology.

